# The relationship between body mass and peak and mean plantar pressure

**DOI:** 10.1186/1757-1146-4-S1-P2

**Published:** 2011-05-20

**Authors:** John Arnold, Ryan Causby, Sara Jones, Hayley Uden

**Affiliations:** 1 School of Health Sciences, Division of Health Sciences, University of South Australia, North Terrace, Adelaide, South Australia, Australia

## Background

The correlation between foot and ankle pathologies and plantar pressure variables has previously been documented in the literature. One of the premises underlying this correlation is the role of increasing body mass on the magnitude of forces acting upon the foot in locomotion, such as the normal force measurable with plantar pressure equipment. Previously, only a limited number of studies have investigated this relationship in the form of plantar pressure variables such as peak and mean pressure in non-pathological adult populations during walking. This study aimed to investigate the effect of increasing body mass added at known intervals on peak and mean plantar pressure variables in healthy adult subjects during walking.

## Methods

In this study, 30 asymptomatic adult subjects were recruited. Additional body mass was simulated via the addition of a weighted vest, with three different loading conditions (5, 10 and 15 kg) including a control condition (0kg). The order of the loading conditions was randomized for each subject. Each subject wore the vest under each loading condition whilst walking down a 10 metre walkway where the outcome variables were measured from 3 steps of the right foot using the mid-gait protocol. The F-Scan in-shoe plantar pressure measurement device (TEKSCAN, Boston, MA, v. 1.6x) was used to collect pressure variables from each subject. The primary outcome variables collected were peak and mean pressure for each of the six plantar regions. A random effects mixed model was used for analysis, including post-hoc pairwise comparisons. Bonferroni adjustments were applied to account for multiple comparison testing.

## Results

Body mass was found to be a significant determinant of peak plantar pressure within the heel, metatarsals 2-5 and hallux regions and within all plantar regions for the mean pressure variable (p < 0.05). Different plantar regions of the foot displayed varying degrees of sensitivity in regards to peak pressure as a response to increasing body mass. The heel and metatarsal 2-5 regions were sensitive to a 10 kg increase in body mass, followed by the metatarsal 1 and hallux regions which only displayed statistically significant increases in body mass when 15 kg of load was applied (p < 0.05). The midfoot and lesser digits did not display any statistically significant differences in peak pressure for all load conditions compared to the control condition. The mean plantar pressure variable displayed similar sensitivity to increasing body mass, with the metatarsal 2-5 region showing significant differences between the 5, 10 and 15 kg loads compared to the control condition (p < 0.05). All other plantar areas were less sensitive, requiring 15 kg of additional load to be added before displaying statistically significant differences in mean pressure compared to the control condition.

## Conclusions

The results of this study indicate an inconsistent relationship between body mass and both peak and mean plantar pressure variables – one that is dependent upon the plantar region.

